# Early Functional Results of Surgery for Organizing Phase of Empyema Thoracis in a High Output Centre for Thoracic Surgery

**DOI:** 10.7759/cureus.12404

**Published:** 2020-12-31

**Authors:** Nadir Ali, Tanveer Ahmad, Khalil A Shaikh, Shagufta Nasreen, Misauq Mazcuri, Ambreen Abid

**Affiliations:** 1 Thoracic Surgery, Jinnah Postgraduate Medical Centre, Karachi, PAK; 2 Thoracic Surgery, Jinnah Postgraduate Medical Center, Karachi, PAK

**Keywords:** early functional results, organized empyema thoracis, pulmonary decortication, entrapped lung

## Abstract

Objective: To determine the early functional outcome of pulmonary decortication (PD) in patients having organized empyema thoracic (ET).

Methodology: This is a prospective study conducted at the Department of Thoracic Surgery, Jinnah Postgraduate Medical Center, Karachi from July 2019 to June 2020. The study included 76 patients referred to the department for assessment and surgery for the indication of organized empyema thoracis. After careful assessment and evaluation of the patients’ computed tomography (CT) scans and pulmonary function tests (PFTs) in addition to their symptoms, history, etiology of empyema, physical examination and nutrition status, they were recommended pulmonary decortication to release the underlying entrapped lung. Functional improvement was measured in terms of forced vital capacity (FVC) and forced expiratory volume in one second (FEV_1_) three to six months after PD.

Results: A total of 76 empyema thoracis patients were included in the study. The mean age of participants was 33.4±11.9 years. The mean duration of empyema symptoms was 7.21±3.7 months. Majority were males (n=61; 80.3%). The full-lung expansion was achieved in 43 patients and partial lung expansion was achieved in 27 patients. The lung failed to expand in five patients. There was one death (lung failed to expand) due to respiratory failure as a result of septicemia. Most of the patients who achieved full-lung expansion had tuberculosis (26; 60.5%), followed by penetrating lung injury (7; 16.2%) and ruptured pulmonary hydatid cysts (5; 11.6%). Statistically significant association was found between etiology and full-lung expansion (p=0.042). Early functional improvement was seen in all patients with PD as mean FEV_1_ improved from 1.23±0.27 to 2.02±0.5 (63% increase; p<0.001) and FVC from 2.10±0.27 to 2.72±0.41 (29.7% increase; p<0.001).

Conclusion: Based on the results obtained in the present study, it is concluded that pulmonary decortication in carefully selected patients has a vital role in significantly improving early functional results in terms of pulmonary functions.

## Introduction

Empyema thoracic (ET) is defined as the presence of pus in the pleural cavity [1,2]. It can arise as a complication of pneumonia or as a result of penetrating thoracic injury, a postoperative complication of lung surgery, perforation of the esophagus either iatrogenic or due to thoracic trauma, thoracic duct injury, or as a complication of thoracentesis and tube thoracotomy [3]. It can also arise as a consequence of pulmonary tuberculosis when a broncho-pleural fistula develops and direct contamination of pleural space occurs with mycobacterium tuberculosis [4].

The definitive surgical treatment in cases of organized ET is pulmonary decortication (PD), whereby, the thick fibrous tissue layer overlying the lung, the diaphragm and the parietal pleura is removed. The goal of this procedure is to release the underlying lung and help re-expand it. Once the fibrous peel is removed from the lung and the chest wall, lung compliance improves, and the lung re-expands, greatly improving the patient’s symptoms and exercise tolerance [3].

If pleural space is breeched by any mechanism be it accidental, diagnostic or therapeutic, serious consequences may result, causing fluid and cellular imbalances and disturbances as well as bacteriological inoculation [2,5]. If infection ensues in the pleural space, ET results in significant medical and surgical consequences for the patient. Over a period of days and weeks, the collection in the pleural space increases with the patient developing signs and symptoms of ET, namely fever, difficulty breathing and fatigue. If it is not managed in a timely manner, ET progresses through the early stages of exudative and fibrinopurulent stages where loculations and adhesions develop, to the third stage, the organizing chronic empyema stage (after three weeks) [3]. Once the lung is entrapped, visceral pleural fibrosis prevents lung expansion. There is thick pus present in the pleural cavity and drug penetration is poor. This leads to sub-therapeutic levels of antibiotics reaching the pleural cavity and the bacteria develop resistance against antibiotic treatment [6].

Even at this stage, a tube thoracostomy is needed to remove the thick pus and reduce the burden of sepsis. Once the patient is fit to undergo a PD procedure, the underlying lung and the contralateral lung should be examined in more detail with the help of a computed tomographic (CT) scan of the chest with contrast [2]. Any cavitatory disease, bronchiectasis, or diffuse parenchymal disease of the entrapped lung should be excluded before this major undertaking as the success of the procedure requires that the underlying lung is devoid of gross disease. Any debilitation of the patient, organ dysfunction, major sepsis or bleeding abnormalities are regarded as contraindications for the procedure.

In the past, studies have reported no improvement in functional outcomes in terms of spirometry for patients undergoing PD [7,8]. Recent studies have supported the importance of PD in terms of improvement of spirometric parameters in terms of forced vital capacity (FVC) and forced expiratory volume in one second (FEV1) [9-12].

This study was designed to evaluate the early (three to six months) functional results of PD in patients having organised ET and assess any improvement in spirometric lung function in terms of FVC and FEV1.

## Materials and methods

**Figure 1:**
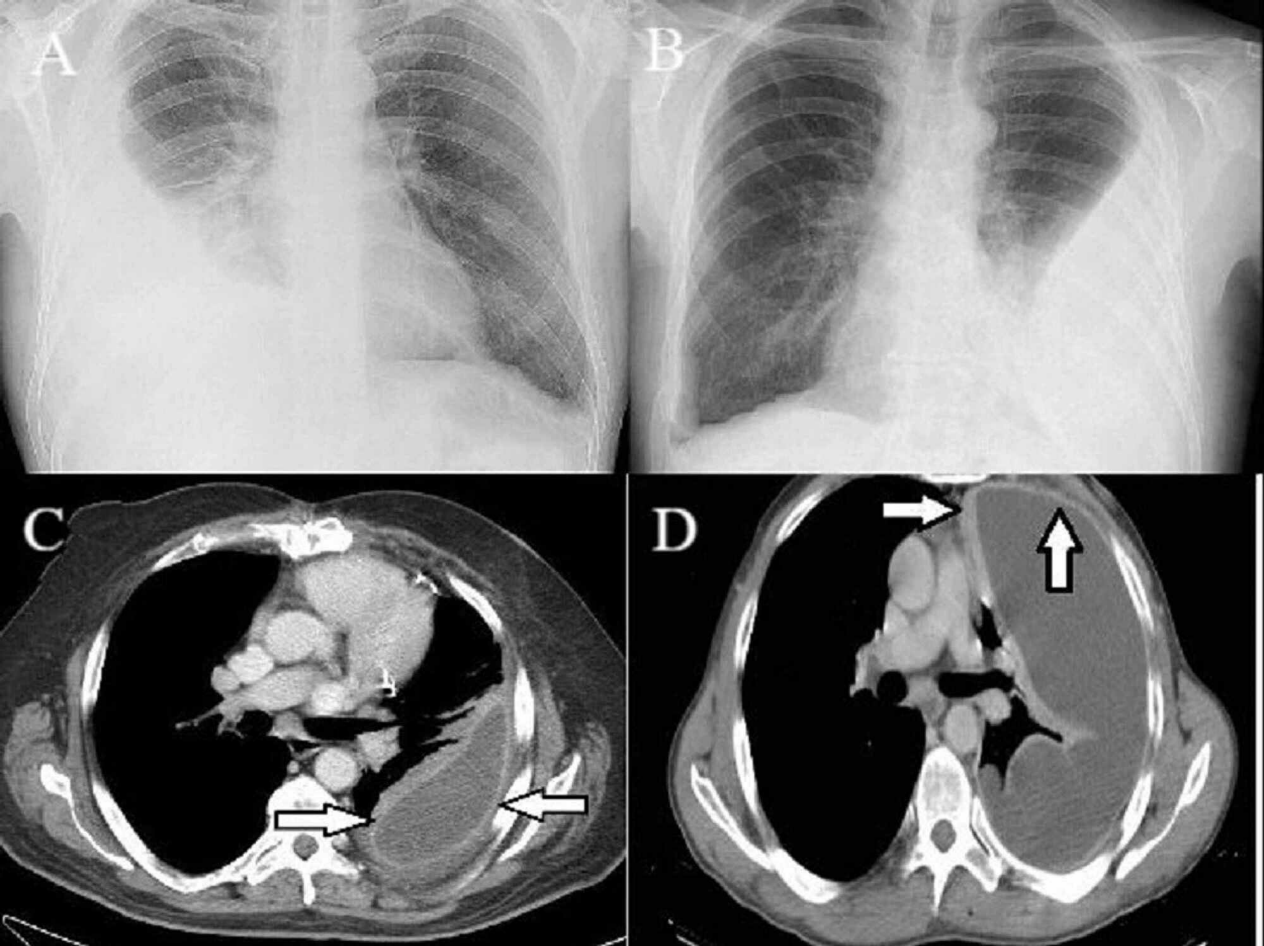
(A) Radiographic findings of empyema thoracis; (B) X-ray of empyema thoracis; (C) and (D) split pleura sign depicting chronicity marked with arrows on CT

This was a prospective study conducted at the Department of Thoracic Surgery, Jinnah Post Graduate Medical Centre (JPMC), Karachi from July 2019 to June 2020. Seventy-six patients aged 15 years and above with more than three weeks history of symptoms of organized empyema thoracis with or without intercostal tube drainage and evidence of split pleura sign on CT scan of the chest with contrast were included in the study (Figure 1).

The initial procedures like thoracentesis or tube thoracostomy to help the lung expand in the fibrino-purulent stage were documented in each case. Bronchoscopy to exclude an endo-bronchial cause of failed lung expansion was done in each case before the definitive procedure. All patients who were not fit for general anaesthesia with a double-lumen endotracheal tube (ETT) were excluded from our study. Any ongoing systemic sepsis, severe debilitation, lung destruction (ipsilateral or contralateral), pulmonary or extra-pulmonary malignancy, or significant organ dysfunction were documented as contra-indications for the procedure.

Prior approval of the ethical committee of JPMC, Karachi was obtained for conducting this study. Patients were informed of the benefit of surgery in their particular cases and their long-term functional prognosis. Informed written consent was obtained from patients or their caregivers. A focused history and physical examination were done and relevant investigations like pus culture/sensitivities, preoperative spirometry and CT chest with contrast were arranged in addition to the baseline investigations and viral markers. Patients were optimized for surgery with the proper administration of antituberculous therapy (ATT) where indicated.

In all cases, we used a 28 or 32 Fr left-sided double-lumen ETT to achieve single lung ventilation for preventing cross-contamination and control of lung movement. In order to adequately remove the thick fibrous peel from the lung, the diaphragm and the chest wall, a muscle cutting incision was utilized. Before closure, all air leaks and bleeding was controlled. It was ensured that all segments of the lung expanded adequately and the pleural space was drained using an underwater seal. In selected cases, we employed high-volume, low-pressure suction to a pressure of −15 to −20 cm with the help of a three-chambered bottle (Atrium®) to help drain the pleural fluid and air.

SPSS version 23 (IBM Corp., Armonk, NY) was used to analyze data. Descriptive analysis was performed for all numeric and categorical variables. The Fisher-exact test was applied to compare functional outcomes with causes of ET. P-value ≤ 0.05 was taken as statistically significant.

## Results

A total of 76 empyema ET patients were included in the study. The mean age of the study participants was 33.4±11.9 years. Sixty-one (80.3%) patients were males whereas 15 (19.7%) were females. The mean duration of symptoms was 7.21±3.7 months (range= 1-17 months).

**Figure 2:**
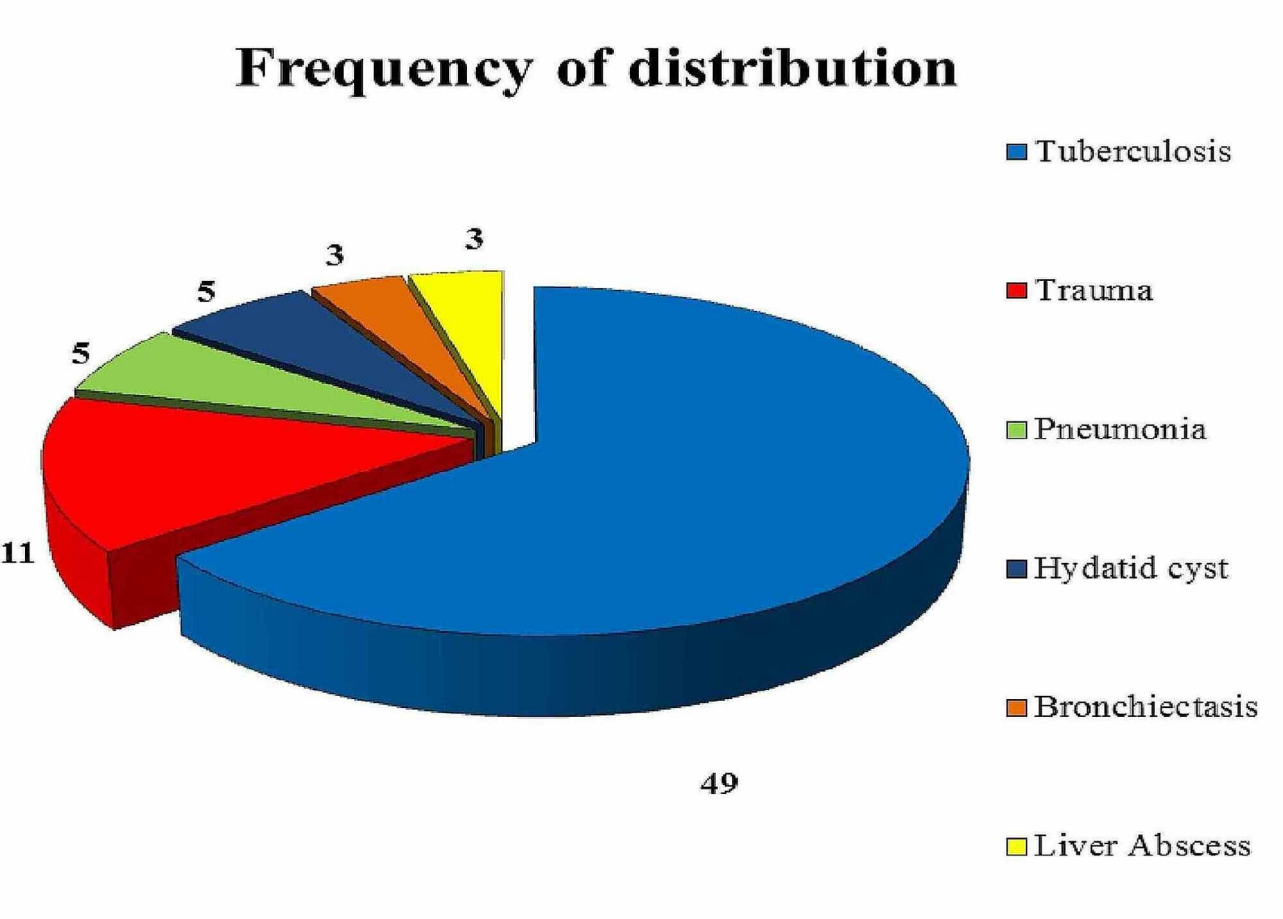
Pie chart representing the frequency of distribution

Forty-nine (64.4%) patients had empyema due to pulmonary tuberculosis (TB) while 11 (14.47%) patients had developed empyema secondary to penetrating chest trauma. Five (6.57%) patients developed empyema as a complication of pneumonia and in another five (6.57%) patients the cause was ruptured pulmonary hydatid cyst. Bronchiectasis led to empyema in three (3.94%) patients and in another three (3.94%) ruptured liver abscess was the cause (Figure 2).

Full lung expansion was achieved in 43 (56.6%) patients; partial lung expansion was achieved in 27 (35.5%) patients and lung failed to expand in only five patients (6.58%) having tuberculosis. One patient (1.32%) died due to sepsis.

The impact of age on early functional outcome was also determined. The mean age of patients showing failure to expand the lung was 45 years (40-52 years), while full-lung expansion was seen in patients with a mean age of 30.4 years (15-70 years). Partial lung expansion was seen in the age group with a mean of 36.47 years (18-50 years).

Other co-factors of significant impact on early lung expansion were smoking and chronic obstructive pulmonary disease (COPD). There were 33 recent smokers in the study. Among them full, partial and failure to expand lung was seen in 13 (39.4%), 15 (45.5%) and 5 (15.2%) patients, respectively. In contrast to this, the rates seen among non-smokers were 33 (76%), 6 (14%), and one (2.3%), respectively. There were 21 patients in the study with COPD. Among them, the rates of full, partial and failure to expand the lung were 6 (28.6%), 11 (52.4%) and 4 (19%), respectively. In contrast, the corresponding rates among patients without COPD were 43 (78.2%), 10 (18.2%) and two (3.63%), respectively. Lung expansion post-PD also showed a statistically significant relationship (p≤0.001) with the duration of chronic ET and symptoms (Table 1).

**Table 1:** Relation of the duration of empyema with functional outcomes (n=76)

Functional outcomes		Duration of empyema			p-value
		1-6 months	7-12 months	>12 months	
Full expansion	Yes	35(94.6%)	14 (43.8%)	0	0.001
	No	2 (5.4%)	18 (56.3%)	7 (100%)	
Partial expansion	Yes	2 (5.4%)	15 (46.9%)	4 (57.1%)	0.001
	No	35 (94.6%)	17 (53.1%)	3 (42.9%)	
Failure to expand	Yes	0	3 (9.4%)	3 (42.9%)	0.001
	No	37 (100%)	29 (90.6%)	4 (57.1%)	

Twenty-one patients who had partial lung expansion were managed by Heimlich® unidirectional valve. The mean duration of lung expansion post valve was 21.09±6.05 days. Once the Heimlich valve was connected, 18 patients with tuberculosis and two patients with trauma had significant lung expansion to improve functional status and exercise tolerance, while one patient with post-pneumonia empyema failed to expand his lung. Two patients (one chest trauma and one tuberculous) had early lung expansion (within 14 days) whereas 18 patients (six chest trauma, six tuberculous, two pneumonia, two pulmonary hydatid cyst rupture, one bronchiectasis and one liver abscess) had late lung expansion (more than 14 days). In all the six (five tuberculous, one pneumonia) patients whose lungs completely failed to expand even after a follow-up of two to four weeks, an open thoracostomy window was created and those patients are in the follow-up phase. The FVC mean values significantly increased after PD from 2.10±0.27 to 2.72±0.41 (p=0.001). Similarly, FEV1 mean values significantly increased from 1.23±0.27 to 2.02±0.55 post-pulmonary decortication. The change in percentage in the postoperative FVC and FEV1 was calculated as 29.8% and 63.4% increase, respectively (Table 2).

**Table 2:** Comparison of pre- and postoperative spirometry parameters FVC: forced vital capacity, FEV_1_: forced expiratory volume in one second.

Spirometry parameters	Pre		Post		% Change	p-value
	Mean	SD	Mean	SD		
FVC	2.101	0.273	2.726	0.414	29.8	0.001
FEV1	1.239	0.278	2.024	0.550	63.4	0.001

Spirometric values were also compared between a tuberculous and non-tuberculous patient with those having full-lung expansion and partial-lung expansion (Table 3).

**Table 3:** Detailed statistical analysis of improvement in spirometric parameters in non-tuberculous and tuberculous patients

Spirometry parameters	Pre		Post		% Change	p-value
	Mean	SD	Mean	SD		
Full-lung expansion	Tuberculosis patient					
FVC	2.07	0.24	2.64	0.39	27.3	0.001
FEV1	1.18	0.2	1.94	0.54	64.4	0.001
Full-lung expansion	Non-Tuberculosis patients					
FVC	2.15	0.33	2.88	0.41	34	0.001
FEV1	1.35	0.36	2.19	0.53	61.7	0.001
Partial-lung expansion	Tuberculosis patients					
FVC	2.07	0.23	2.45	0.33	18.2	0.001
FEV1	1.19	0.16	1.58	0.44	32.8	0.001
Partial-lung expansion	Non-tuberculosis patients					
FVC	2.12	0.30	2.89	0.37	36.2	0.001
FEV1	1.28	0.32	2.29	0.41	79.6	0.001

## Discussion

Earlier studies have shown arguably, that there is no significant improvement in the functional status (spirometry results) of chronic ET patients undergoing PD [7,8]. Recent studies have, however, supported the importance of PD as the cornerstone intervention in the effective management of chronic ET both in tuberculous and non-tuberculous patients [9-15]. Abraham and Chikkaahonnaiah have concluded from their recent study that both FEV1 and FVC significantly improved by 14% and 17% similar to what our results have shown [12]. Before PD is attempted, it is most important that the empyema cavity is made free of the purulent, thick pus with the help of a large calibre chest tube. Furthermore, judicious use of indicated antibiotics plays a vital part in reducing the burden of sepsis. Once the pus is removed, the empyema cavity can be managed with PD with much less morbidity [16].

Among our patients most presented with some degree of chest cavity asymmetry and contraction. This asymmetry was more significant among patients with a longer duration of symptoms (more than seven months). Gokce et al. have described their results of PD in 33 patients of organized ET in whom there were statistically significant improvements seen in the transverse and antero-posterior diameters of the chest after surgery [13]. We have also observed similar improvements (68/76; 89.5%) in our patients after PD. This resulted in both cosmetic and functional improvements in terms of posture and exercise tolerance. The affected side of the chest was significantly smaller due to the pleural contraction and overcrowding of ribs with resultant narrowing of intercostal space. Furthermore, there was universal shoulder drooping on the affected side (more pronounced in patients with long-term chest tube insertion). These findings have been reported by other authors who hypothesized a worse postoperative result in terms of spirometric parameters. Surprisingly, the postoperative results were not statistically significant [2,13]. Our results have also shown that after successful PD, pulmonary function tests (PFTs) improve remarkably within three to six months of surgery even with clinical and radiological chest cavity contraction and the patient is able to tolerate climbing stairs without getting out of breath and in a few weeks is able to earn a living.

In our patients, we were confronted with tuberculosis as the most common (49/76; 64.5%) cause of organized ET. Tuberculosis is still very commonly seen in the local community. It is poorly managed in terms of early diagnosis and treatment, and hence, the majority of patients in this study comprised of organised ET due to TB. In another study from India, a similar incidence (60%) of tuberculous ET was reported recently. Functional results of PD after carefully selecting patients with tuberculous ET have shown variable improvement from the preoperative values [12]. A study by Gokce et al. including 11 (33% of the total number) patients with organised tuberculous ET also substantiates our results in terms of improved exercise endurance status [13]. Although the results obtained by the authors did not achieve a statistical significance, both FEV1 and FVC did improve and patients' exercise tolerance was much better after PD. Similarly, other authors have seen a significant increase in the functional spirometric values after PD in tuberculous ET patients [9,10]. The decision to go ahead with PD is not to be taken lightly as there are many factors that will lead to a failed attempt at PD. Patients' nutritional status, the condition of the underlying lung, any cavitatory disease, active tuberculosis, or not enough ATT treatment are some of the factors that can result in a failed PD procedure and a failure in achieving adequate functional improvement in spirometric parameters [10].

The other common causes of organized ET were secondary to penetrating injury of the chest (11; 14.5%), postpneumonic empyema (5; 6.6%), empyema due to ruptured hydatid cyst (5; 6.6%), liver abscess (3; 3.9%) and bronchiectasis (3; 3.9%). These patients were referred to us often quite late (lapse of 30-90 days) when all measures to resolve the condition short of PD had already been tried and failed. Initial management of tube thoracostomy due to hemo-pneumothorax had already been carried out at the peripheral primary centre often ill-equipped to handle these acute cases. Some of the problems identified with the tube thoracostomies were inadequate calibre, blocked tubes, inadequate seal around the tube thoracostomy, infected dressing oozing thick purulent pus, maggots visible in the wound and tube and inadequate seal on the underwater seal bottles as identified by a study by Ahmad et al. When referred to us, all patients had well established organized ET [17]. They had lost weight (average of 6 kg) and their tube thoracostomies had not been taken care of.

Poorly treated community-acquired pneumonia (CAP) is a well-established cause of ET [18]. Most of the patients in our study were middle-aged (50 to 70) with a history of late diagnosis and inadequately treated pneumonia. On further investigations, 20% of the patients had uncontrolled diabetes mellitus (HBA1C= 9-13 mmol/L). Organized ET was our working diagnosis in these patients supported by pleural fluid analysis and CT chest findings. Investigations with anthropometric measurements were done in all cases to determine their suitability for surgical decortications. CT scan was obtained to define the extent of their disease and to establish any underlying parenchymal pathology detrimental to PD. CT scans confirmed organized ET due to the presence of split pleura sign, chest wall contraction and overcrowding of ribs [19].

We have managed chronic ET due to the rupture of pulmonary hydatid cysts in five patients in the present study. Rupture of the cyst usually happens due to iatrogenic reasons [20]. Two patients had a history of ultrasound aspiration of the cyst while under treatment in interior Sindh. In another three patients, no such history was available and a spontaneous rupture was presumed [20].

In the present study, postoperative complications were seen in 20 (26.3%) patients. Among these, the most significant were postoperative bleeding (n=6), prolonged air leak of seven or more days (n=5), atrial fibrillation (n=3), and residual collections (n=6). All complications were managed aggressively and patients were followed up for a mean duration of 6.5 months (range 4-12 months). There was one (1.32%) mortality in this study. A 60-year-old man with postpneumonic chronic ET did not survive surgery and died on the fourth day of surgery due to respiratory failure. Our mortality rate due to PD is in agreement with earlier similar studies reporting this to be between 1.3 and 6.6% [21].

*Strengths of the study*:A reasonable number of patients in the study; it is a prospective data collection study and all patients were operated on by the same surgeon. *Weakness of the study*: The study is a single-center study; the use of only FVC and FEV1 as measures of pulmonary function.

## Conclusions

Based on the results obtained in the present study, it is concluded that pulmonary decortication in carefully selected patients with chronic empyema thoracis plays a definitive role in significantly improving early functional results in terms of pulmonary functions (FVC and FEV1). It also improves the physical posture of the patient and improves exercise tolerance.
